# Tocilizumab does not block interleukin-6 (IL-6) signaling in murine cells

**DOI:** 10.1371/journal.pone.0232612

**Published:** 2020-05-04

**Authors:** Juliane Lokau, Florian Kleinegger, Yvonne Garbers, Georg H. Waetzig, Joachim Grötzinger, Stefan Rose-John, Johannes Haybaeck, Christoph Garbers

**Affiliations:** 1 Department of Pathology, Medical Faculty, Otto-von-Guericke-University Magdeburg, Magdeburg, Germany; 2 Diagnostic & Research Center for Molecular BioMedicine, Institute of Pathology, Medical University of Graz, Graz, Austria; 3 Institute of Psychology, Kiel University, Kiel, Germany; 4 CONARIS Research Institute AG, Kiel, Germany; 5 Institute of Biochemistry, Kiel University, Kiel, Germany; 6 Department of Pathology, Neuropathology and Molecular Pathology, Medical University of Innsbruck, Innsbruck, Austria; Texas Woman's University, UNITED STATES

## Abstract

Tocilizumab is a humanized monoclonal antibody that is approved for the treatment of different human inflammatory diseases, including rheumatoid arthritis and cytokine release syndrome. Tocilizumab binds to the interleukin-6 receptor (IL-6R) and thereby blocks signaling of the pro-inflammatory cytokine IL-6. Initial studies and all authority assessment reports state that tocilizumab is effective in humans, but cannot bind to the murine or rat IL-6R and thus not block IL-6 signaling in the mouse. However, several recent studies described the use of tocilizumab in mice and reported biological effects that were attributed to IL-6 blockade. In this study, we investigate the capability of tocilizumab to block IL-6 signaling using different human and murine cell lines. Our results unequivocally confirm the original state of the art that tocilizumab blocks signaling via the human IL-6R, but does not block IL-6 signaling in murine cells.

## Introduction

Interleukin-6 (IL-6) is a cytokine with well-described pro-inflammatory functions. IL-6 is barely detectable in healthy individuals, but strongly produced in nearly all inflammatory diseases, where it has been identified as a key factor for their initiation, development and/or persistence [1]. IL-6 binds to the IL-6 receptor (IL-6R), which is expressed on hepatocytes and several leukocyte subsets, and initiates signaling through a homodimer of the ubiquitously expressed signal-transducing co-receptor gp130. Because gp130 is shared with other cytokines of the IL-6 family [2], IL-6 and the IL-6R have been the primary targets for specific therapeutic interventions against detrimental IL-6 functions [3].

Several monoclonal antibodies directed against IL-6 or IL-6R are already in clinical use, and many more are currently under development [3]. The first marketed drug is tocilizumab, a humanized monoclonal antibody that binds to the cytokine-binding module (CBM) of the IL-6R that is located in its domains D2 and D3. This prevents binding of IL-6 to the IL-6R via its so-called *site I*. Tocilizumab is approved for the treatment of patients with rheumatoid arthritis, juvenile idiopathic arthritis, Castleman’s disease, giant cell arteritis, and cytokine release syndrome [3].

IL-6 shows some species-specific features. While human IL-6 can bind to both the human and the murine IL-6R, murine IL-6 can only bind to the murine, but not the human IL-6R [4, 5]. Furthermore, there are important structural differences between the human and the murine IL-6R [6]. Tocilizumab does not cross-react with mouse or rat IL-6R [7–9], and a genetically humanized mouse had to be generated to enable the analysis of anti-human IL-6R therapeutics in the mouse [10]. Accordingly, a surrogate antibody analogous to tocilizumab that blocks IL-6 signaling in the mouse and rat (MR16-1) had to be used for the pre-clinical studies in rodents that were required for the approval of tocilizumab (see, e.g. [11]).

Surprisingly, four studies published in 2018 and 2019 used tocilizumab in different murine disease models to block IL-6 signaling in the mouse and claimed IL-6-specific effects [12–15]. They report findings on different disease models like perioperative neurocognitive disorder [12], ischemic osteonecrosis [13], juvenile diabetes [14] and diabetic nephropathy [15] and attribute the observed effects to a successful blockade of IL-6 signaling. This means that either the knowledge that tocilizumab does not block IL-6 signaling in the mouse is not present anymore in parts of the scientific community, or that researchers have previously overlooked that tocilizumab can block IL-6 signaling via the murine IL-6R. This alarmed us to re-assess the capability of tocilizumab to block murine IL-6 signaling.

## Materials and methods

### Cells and reagents

Ba/F3-gp130-hIL-6R cells (expressing the human IL-6R) and Ba/F3-gp130-mIL-6R cells (expressing the murine IL-6R) have been described previously [8]. The human monocyte cell line U-937 (CRL-1593.2) and the murine macrophage cell line RAW264.7 (SC-6003) were obtained from the American Type Culture Collection (ATCC, Manassas, VA, USA), and the human hepatocyte cell line HepG2 (ACC 180) was purchased from the German Collection of Microorganisms and Cell Cultures (DSMZ, Braunschweig, Germany). The murine hepatocyte cell line AML-12 was provided by the Institute of Pathology of the Medical University Graz (Graz, Austria). Ba/F3-gp130-hIL-6R, Ba/F3-gp130-mIL-6R, U-937, RAW264.7 and HepG2 cells were cultured in DMEM containing 10% fetal bovine serum (FBS), 60 mg/l penicillin and 100 mg/l streptomycin (all from Sigma-Aldrich, Munich, Germany). For Ba/F3-gp130-hIL-6R and Ba/F3-gp130-mIL-6R cells, 10 ng/ml human IL-6 (hIL-6) or murine IL-6 (mIL-6) were added to the medium. AML-12 cells were cultured in DMEM/Ham’s F12 (Gibco, Thermo Fisher Scientific, Darmstadt, Germany) containing 10% FBS, 60 mg/l penicillin, 100 mg/l streptomycin, 2 mM L-Glutamine, 1% ITS (Gibco/Thermo Fisher Scientific), and 40 ng/ml dexamethasone (Sigma-Aldrich). All cells were kept in a standard incubator at 37°C and 5% CO_2_ in a water-saturated atmosphere. hIL-6 was produced as described previously [8], mIL-6 was purchased from Immunotools (Friesoythe, Germany). Tocilizumab and MR16-1 were kindly provided by Stefan Rose-John. Primary antibodies directed against phosporylated signal transducer and activator of transcription-3 (pSTAT3) (pY705, D3A7), STAT3 (124H6), and β-actin were obtained from Cell Signaling Technology (Frankfurt/M, Germany). The secondary antibodies Alexa Fluor488-conjugated donkey anti-rabbit IgG and Alexa Fluor647-conjugated goat anti-mouse IgG were from Thermo Fisher Scientific. IRDye 680RD-conjugated goat anti-mouse IgG and IRDye 800CW-conjugated donkey anti-rabbit IgG were purchased from LI-COR Biosciences (Bad Homburg, Germany).

### Cell viability assay

IL-6-induced proliferation of Ba/F3-gp130-hIL-6R and Ba/F3-gp130-mIL-6R cells was determined as described previously [8]. In brief 5000 cells per well were cultured in 96-well plates in the presence of IL-6 and different amounts of tocilizumab for 48h. The amount of viable cells was determined using the Cell Titer Blue reagent (Promega, Mannheim, Germany) according to the manufacturer’s instructions. Data are presented as relative light units (RLU) and one experiment (triplicates ± SD) out of three with similar outcome is shown.

#### Stimulation of cells, western blotting and densitometric analysis

Adherent cells (RAW264.7, HepG2, AML-12) were seeded into 6-well plates 24 h prior to the experiment. All cells were washed 3 times with phosphate-buffered saline (PBS) and afterwards starved in serum-free medium for 2 h (Ba/F3-gp130-hIL-6R and Ba/F3-gp130-mIL-6R) or 4 h (all other cell types). The cells were incubated with the indicated amounts of tocilizumab for 30 min and then stimulated with 10 ng/ml hIL-6 or mIL-6 (for human or murine cells, respectively) for 15 min. Subsequently, cells were harvested, lysed, and 40 μg protein were separated by SDS-PAGE and blotted onto nitrocellulose membranes. After blocking, membranes were incubated with primary antibodies at 4°C over night and, after washing, with AlexaFluor-conjugated or IRDye-conjugated secondary antibodies for 1h at room temperature in the dark. Signals were detected using the a ChemoStar ECL Imager (Intas, Göttingen, Germany) or an Odyssey Fc Imager (LI-COR Biosciences, Bad Homburg, Germany), and densitometric analysis was performed using the ImageStudio software (LI-COR Biosciences, Bad Homburg, Germany). Data are presented as ratio of the pSTAT3/STAT3 signals from three independent experiments (mean ±SD). Additionally, one representative western blot per experimental setting is shown.

### Sequence alignment

The amino acid sequences of the human IL-6R (entry #P08887) and the murine IL-6R (entry #P22272) were retrieved from UniProt and aligned using clustal omega (https://www.ebi.ac.uk/Tools/msa/clustalo/). The residues participating in *site I* interactions according to [16] were highlighted.

### Statistical analysis

All analysis were conducted using GraphPad Prism 8 (GraphPad Software, San Diego, CA, USA). Data were analysed with one-way ANOVA and Dunnett's multiple comparisons test.

## Results

### The IL-6/IL-6R interface site I is not conserved between human and murine IL-6R

**Fig 1:**
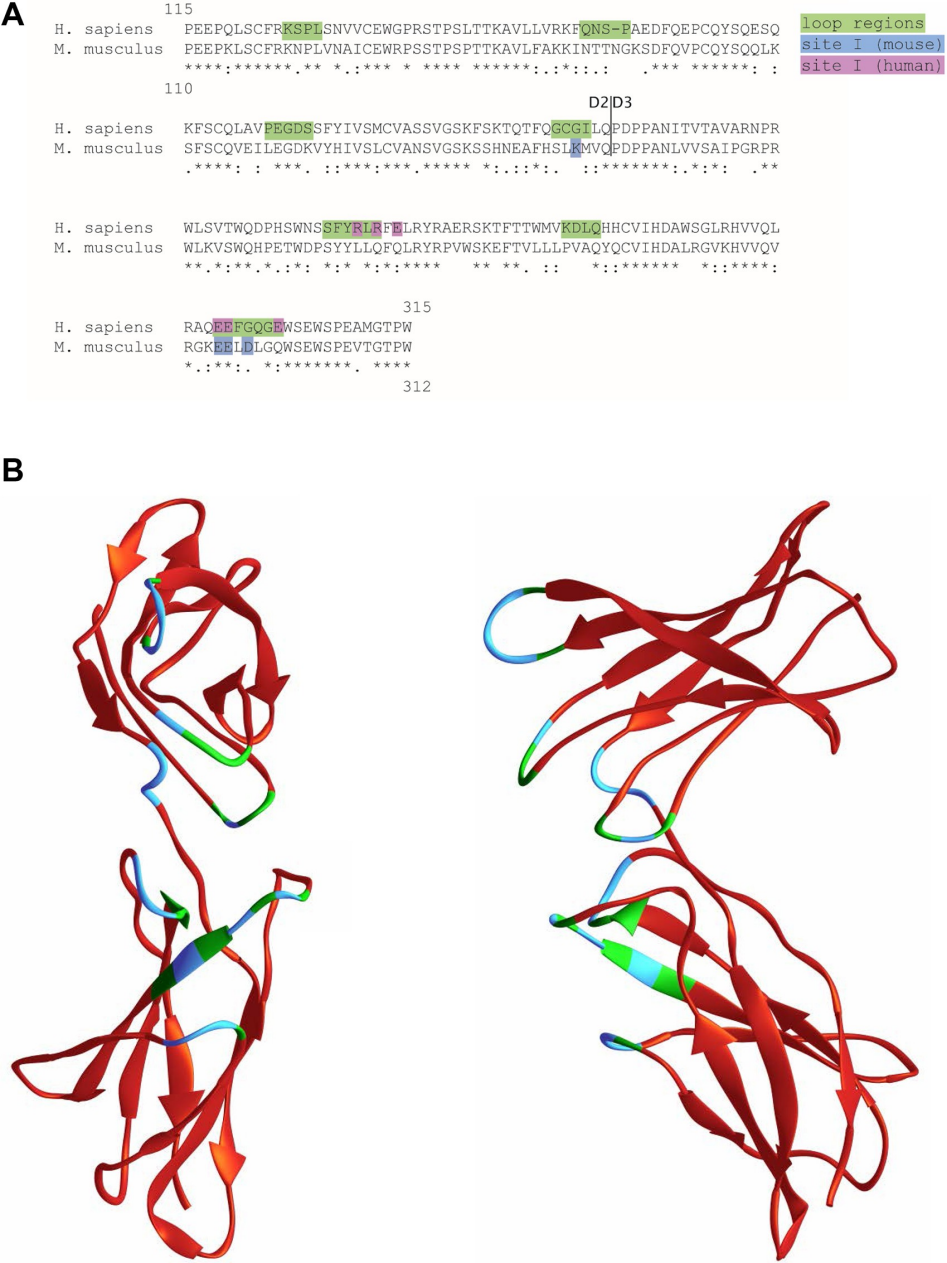
The IL-6/IL-6R interface site I is not conserved between human and murine IL-6R. **(A)** Alignment of the amino acid sequence of the D2 and D3 domains from human and murine IL-6R. Residues previously described to be involved in IL-6 binding are highlighted. **(B)** Ribbon representation of the human IL-6R D2 and D3 domain structure [17, 18]. (left: front view; right: side view). Regions which are in involved in binding to site I of IL-6 are depicted in green. Amino acid residues in these loop regions that are different in the mouse IL-6R are colored in blue.

IL-6 binds to the domains D2 and D3 of the IL-6R, which constitute the CBM. Tocilizumab, which blocks binding of IL-6 to the IL-6R, binds to the same residues on the IL-6R as IL-6. A sequence alignment of the D2 and D3 domains of the human and the murine IL-6R revealed a moderate conservation between the two species (Fig 1A). Importantly, amino acid residues that have previously been identified as involved in the IL-6/IL-6R interaction are neither conserved nor located at the same position within the IL-6R sequence [16]. The only exception are the two adjacent glutamic acid residues at positions 296/297 (human IL-6R) and 293/294 (murine IL-6R) (Fig 1A). Importantly, when we used the structure of the human IL-6R and highlighted the amino-acid residues forming the site I interface, the derivations between human and murine IL-6R became even more obvious (Fig 1B). Thus, this *in silico* analysis already strongly argues against tocilizumab being able to block both human and murine IL-6R.

### Tocilizumab does not block mIL-6-induced cell proliferation or STAT3 phosphorylation in Ba/F3-gp130-mIL-6R cells

**Fig 2:**
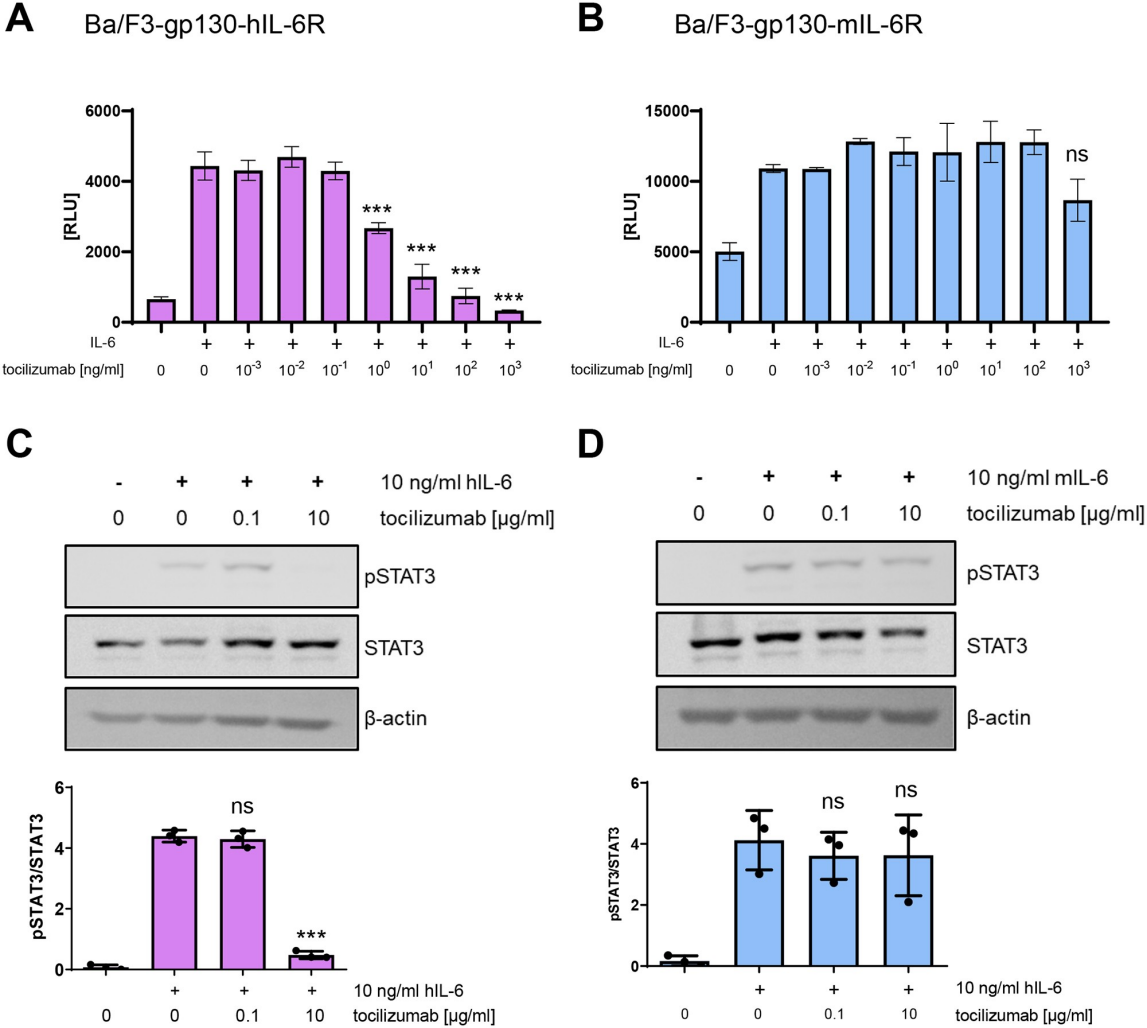
Response of Ba/F3-gp130 cells with human or murine IL-6R to tocilizumab. **(A)** Viability assay of Ba/F3-gp130-hIL-6R cells. Equal amounts of cells were stimulated with hIL-6 and incubated with different concentrations of tocilizumab as indicated for 48 h. Cell viability was measured and is shown in relative light units (RLU). One experiment out of three with similar outcome is shown. Data are shown as mean ± SD (n = 3). **(B)** The experiment was performed as described under (A) but with Ba/F3-gp130-mIL-6R cells and mIL-6. Data are shown as mean ± SD (n = 3). **(C)** Ba/F3-gp130-hIL-6R cells were pretreated with tocilizumab as indicated for 30 min and then stimulated with 10 ng/ml hIL-6 for 15 min. Phosphorylation of STAT3 was determined by western blot. Quantification of three independent experiments and one representative western blot is shown. **(D)** The experiment was performed as described under (C) but with Ba/F3-gp130-mIL-6R cells and mIL-6. Statistical significance was analyzed with one-way ANOVA followed by Dunnett's multiple comparisons test (***: p < 0.001; ns: not significant).

In order to experimentally investigate whether tocilizumab only blocks signaling via the human IL-6R, we used Ba/F3-gp130 cells stably transduced with a cDNA encoding the human IL-6R (termed Ba/F3-gp130-hIL-6R). These cells proliferate only in the presence of hIL-6 and undergo apoptosis otherwise. As expected, tocilizumab was able to block proliferation of Ba/F3-gp130-hIL-6R cells in a dose-dependent manner (Fig 2A). In contrast, when we stably expressed a cDNA encoding the murine IL-6R in Ba/F3-gp130 cells (termed Ba/F3-gp130-mIL-6R), tocilizumab was not able to block proliferation induced by mIL-6 (Fig 2B). We substantiated this finding by analyzing phosphorylation of the key IL-6-targeted transcription factor STAT3. As shown in Fig 2C, 10 μg/ml tocilizumab completely abrogated STAT3 phosphorylation induced by 10 ng/ml hIL-6 in Ba/F3-gp130-hIL-6R cells. In contrast, the same amount of tocilizumab had no influence on STAT3 phosphorylation induced by mIL-6 in Ba/F3-gp130-mIL-6R cells (Fig 2D). These experiments confirm previous results [7–9] and show that tocilizumab is not able to block signaling via the murine IL-6R in Ba/F3-gp130 cells.

### The surrogate antibody MR16-1, an analog to tocilizumab, blocks IL-6 signaling via the murine, but not via the human IL-6R

**Fig 3:**
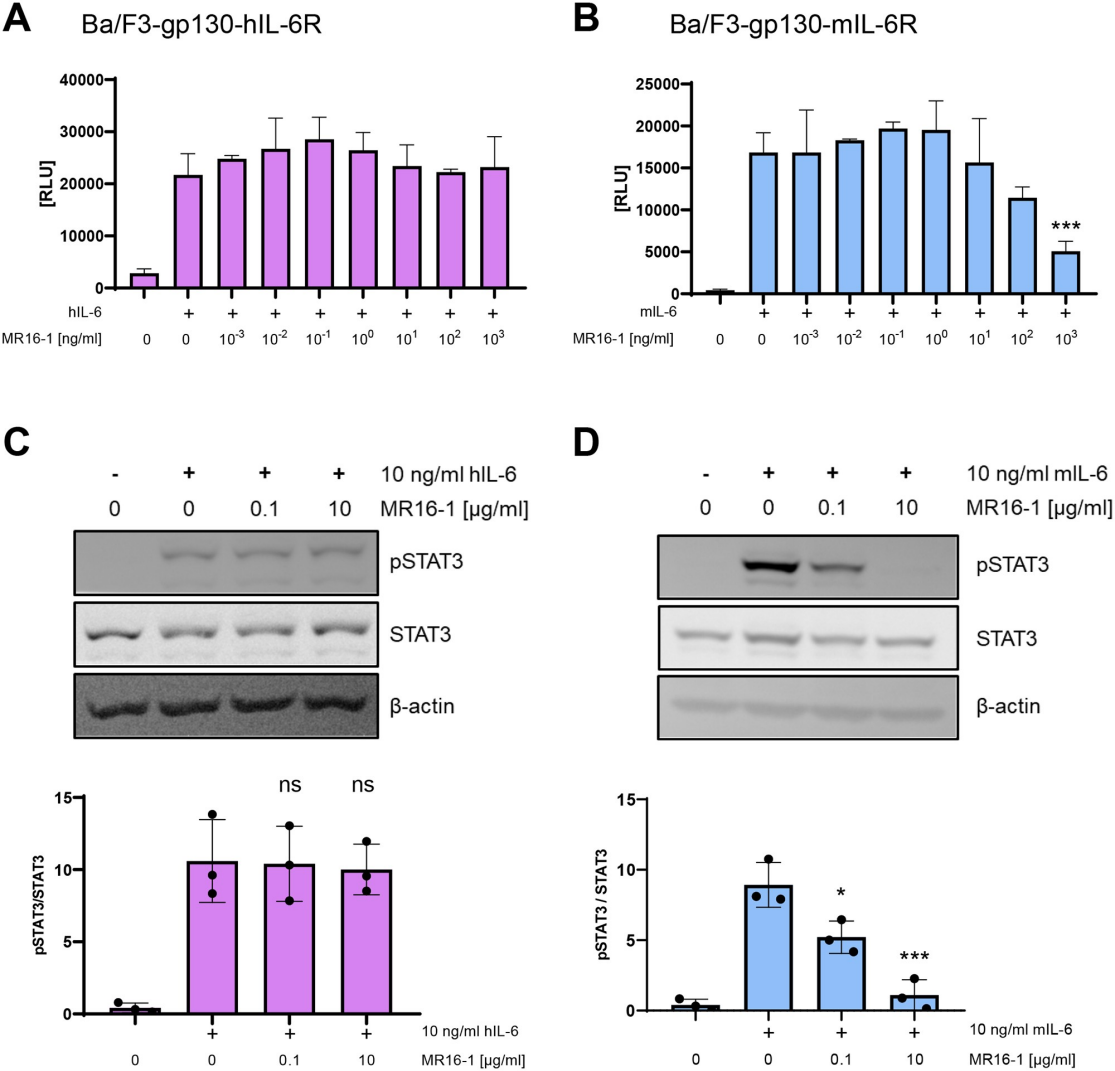
Response of Ba/F3-gp130 cells with human or murine IL-6R to MR16-1. **(A)** Viability assay of Ba/F3-gp130-hIL-6R cells. Equal amounts of cells were stimulated with hIL-6 and incubated with different concentrations of MR16-1 as indicated for 48 h. Cell viability was measured and is shown in relative light units (RLU). One experiment out of three with similar outcome is shown. Data are shown as mean ± SD (n = 3). **(B)** The experiment was performed as described under (A) but with Ba/F3-gp130-mIL-6R cells and mIL-6. Data are shown as mean ± SD (n = 3). **(C)** Ba/F3-gp130-hIL-6R cells were pretreated with MR16-1 as indicated for 30 min and then stimulated with 10 ng/ml hIL-6 for 15 min. Phosphorylation of STAT3 was determined by western blot. Quantification of three independent experiments and one representative western blot is shown. **(D)** The experiment was performed as described under (C) but with Ba/F3-gp130-mIL-6R cells and mIL-6. Statistical significance was analyzed with one-way ANOVA followed by Dunnett's multiple comparisons test (*: p<0.05; ***: p < 0.001; ns: not significant).

In order to exclude that the murine IL-6R in the Ba/F3-gp130-mIL-6R cell line does not react to inhibition by an antibody, we repeated the same experiments with the antibody MR16-1, which is a surrogate antibody analogous to tocilizumab and blocks IL-6 signaling in the mouse. As expected, MR16-1 did not influence cell proliferation of Ba/F3-gp130-hIL-6R cells induced by hIL-6 (Fig 3A), but blocked proliferation of Ba/F3-gp130-mIL-6R cells induced by mIL-6 in a dose-dependent manner (Fig 3B). In line with this, MR16-1 did not prevent the phosphorylation of STAT3 when Ba/F3-gp130-hIL-6R cells were stimulated by hIL-6 (Fig 3C), but efficiently blocked STAT3 phosphorylation in Ba/F3-gp130-mIL-6R cells induced by mIL-6 (Fig 3D). Thus, our results clearly show that signaling via the murine IL-6R can be blocked by the appropriate antibody MR16-1, but that this is not possible with the inappropriate antibody tocilizumab.

### Tocilizumab blocks IL-6 signaling in human, but not murine cells with endogenous IL-6R expression

**Fig 4:**
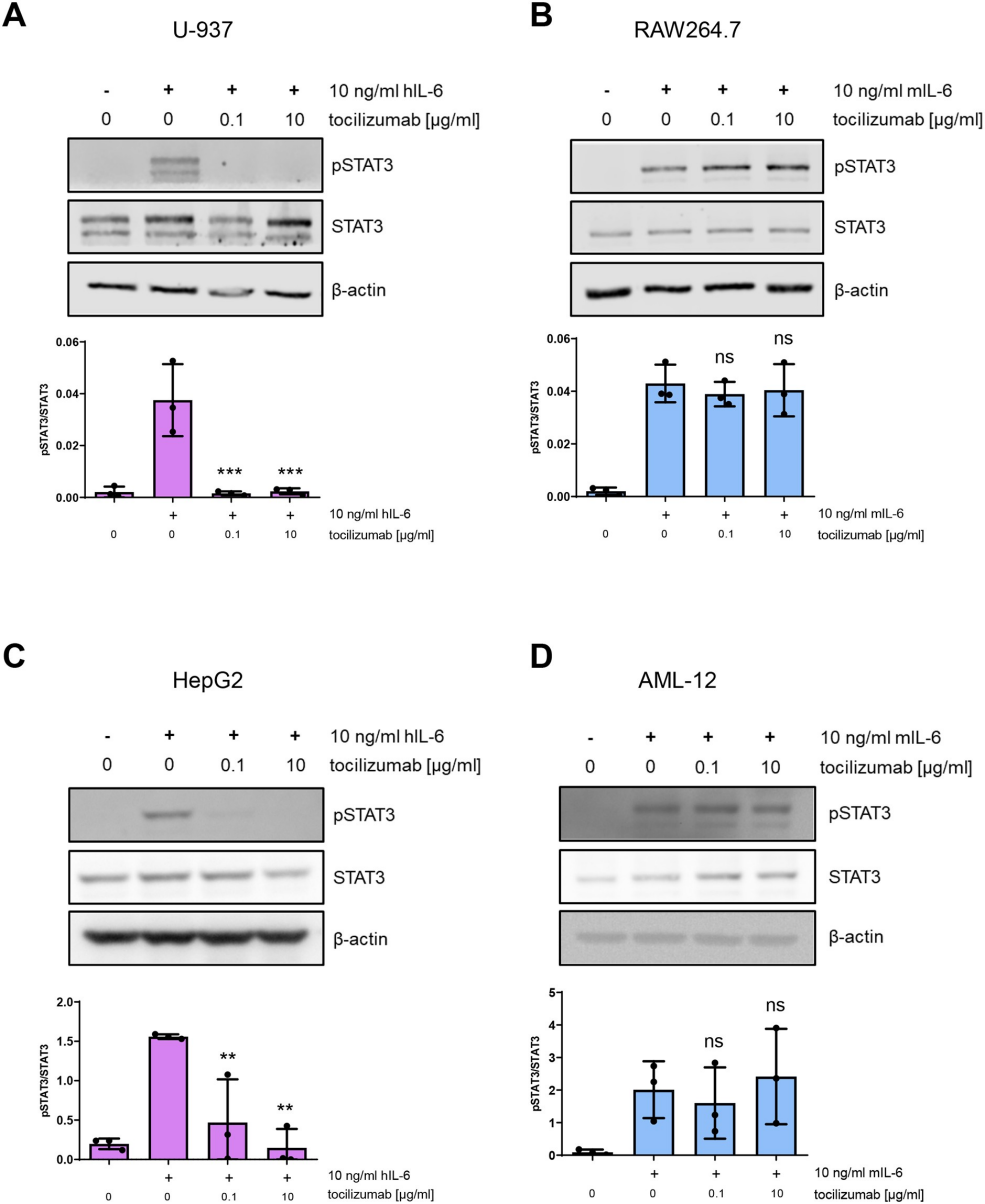
Tocilizumab blocks IL-6 signaling in human, but not murine cells with endogenous IL-6R expression. **(A)** Human U-937 cells were pretreated with tocilizumab as indicated for 30 min and then stimulated with 10 ng/ml hIL-6 for 15 min. Phosphorylation of STAT3 was determined by western blot. Quantification of three independent experiments and one representative western blot are shown. **(B-D)** The experiments were performed as described under (A) but with murine RAW264.7 cells and mIL-6 (B), with human HepG2 cells and hIL-6 (C) and with murine AML-12 cells and mIL-6 (D). Statistical significance was analyzed with one-way ANOVA followed by Dunnett's multiple comparisons test (**: p<0.01; ***: p < 0.001; ns: not significant).

In order to exclude that the overexpression of IL-6R in the Ba/F3-gp130 cells influences the inhibition profile of tocilizumab, we performed similar experiments with cell lines expressing the IL-6R endogenously. Stimulation of U-937 cells, a standard human monocytic cell line originating from a histiocytic lymphoma, with hIL-6 induced robust STAT3 phosphorylation. This was completely abrogated when the cells were pretreated with tocilizumab (Fig 4A). In contrast, tocilizumab had no effect on STAT3 phosphorylation when RAW264.7 cells, a standard murine macrophage cell line, were stimulated with mIL-6 (Fig 4B). hIL-6 induced STAT3 phosphorylation also in HepG2 cells, a human hepatocellular carcinoma cell line. As in U-937 cells, no activation of STAT3 was detected via western blot when the cells were pre-treated with tocilizumab (Fig 4C). We further stimulated the murine AML-12 hepatocytic cell line with mIL-6. As in their hIL-6-stimulated human counterparts, mIL-6 induced STAT3 phosphorylation in the murine AML-12 cells, but even 10 μg/ml tocilizumab were not able to block the biological activity of 10 ng/ml mIL-6 (Fig 4D). In summary, our experiments did not reveal any ability of tocilizumab to block IL-6 signaling via the murine IL-6R, while it efficiently blocked IL-6 signaling on all human cell lines examined.

## Discussion

IL-6 is an important cytokine that has been recognized early on as a suitable therapeutic target in inflammatory human diseases. Consequently, numerous antibodies directed against IL-6 or the IL-6R have been developed and some of them are already approved for clinical use [3]. One of them, tocilizumab, was created in the 1990s and is currently used to treat rheumatoid arthritis in more than 100 countries. Previous studies have shown that tocilizumab is highly active against the human IL-6R, but does not recognize or block the murine IL-6R [7–9]. Because safety studies in rodents are a prerequisite for approval of a therapeutic compound designated for human use, the surrogate antibody MR16-1, which showed an inhibitory capacity comparable to tocilizumab, had to be developed [7].

Astonishingly, several studies that used tocilizumab in different mouse models to block IL-6 signaling and claimed IL-6-specific effects have recently been published [12–15]. However, no direct proof is included in any of these studies showing that IL-6 signaling is indeed blocked in mice injected with tocilizumab, such as inhibition of downstream signaling cascades or a reduction in IL-6-induced target genes. If tocilizumab was actually able to block murine IL-6 signaling, this would have major consequences for future studies addressing IL-6 biology.

In this study, we therefore comprehensively analyzed the capacity of tocilizumab to block IL-6 signaling using different well-characterized human and murine model cell lines of myelomonocytic and hepatic origin, which represent the main target cells of IL-6. Our results unequivocally show that tocilizumab blocks signaling of human IL-6 via the human IL-6R, but has absolutely no capability to block signaling of murine IL-6 via the murine IL-6R. These findings are consistent with the previous state of the art and all regulatory documents for tocilizumab [7–9] and are further supported by an *in silico* analysis, which shows that the binding interfaces of the human and the murine IL-6R towards *site I* of IL-6 are not conserved between the two species.

In conclusion, we find no evidence for an IL-6-blocking activity of tocilizumab in murine cells. The epitope that tocilizumab targets on the human IL-6R is not present on the murine IL-6R, and accordingly, tocilizumab fails to block signaling via the murine IL-6R. Therefore, our results suggest that the recently published effects attributed to tocilizumab in inflammatory mouse models are not directly caused by the blockade of IL-6, but either represent true unspecific effects of the antibody or experimental artifacts.

## Supporting information

S1 Data (PDF)
