# Supplementary material for: Tocilizumab does not block interleukin-6 (IL-6) signaling in murine cells
Source: PLoS One. 2020 May 4;15(5):e0232612. doi: 10.1371/journal.pone.0232612 (PMC7197771; doi:10.1371/journal.pone.0232612)
Supplement: S1 Data — (PDF) [file pone.0232612.s001.pdf]

Figure 2 D/E

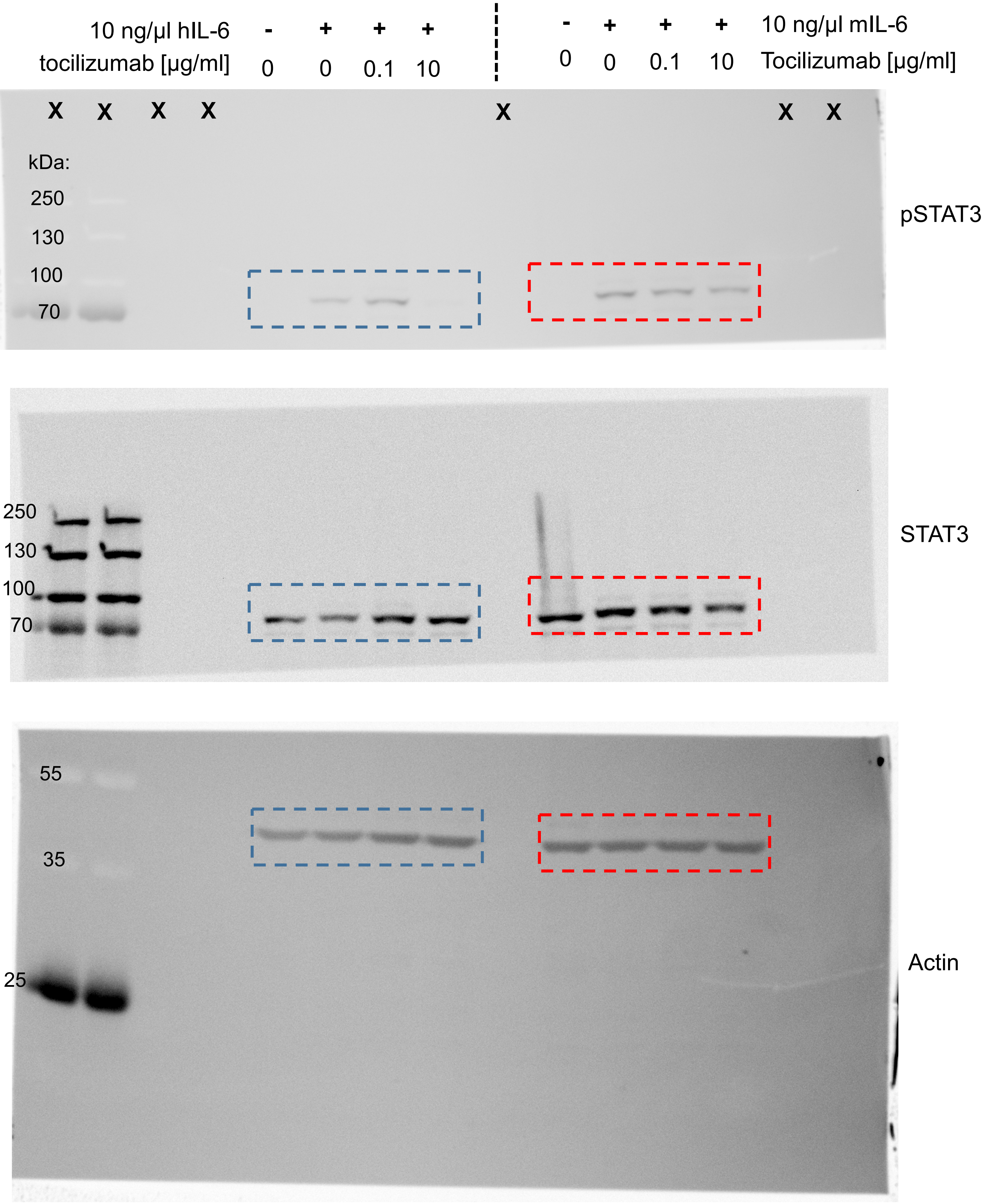

Detected with Alexa Fluor488- and Alexa Fluor647-conjugated antibodies using a ChemoStar ECL Imager (Intas)

Lanes not included in the figures are marked with an X

Figure 3C

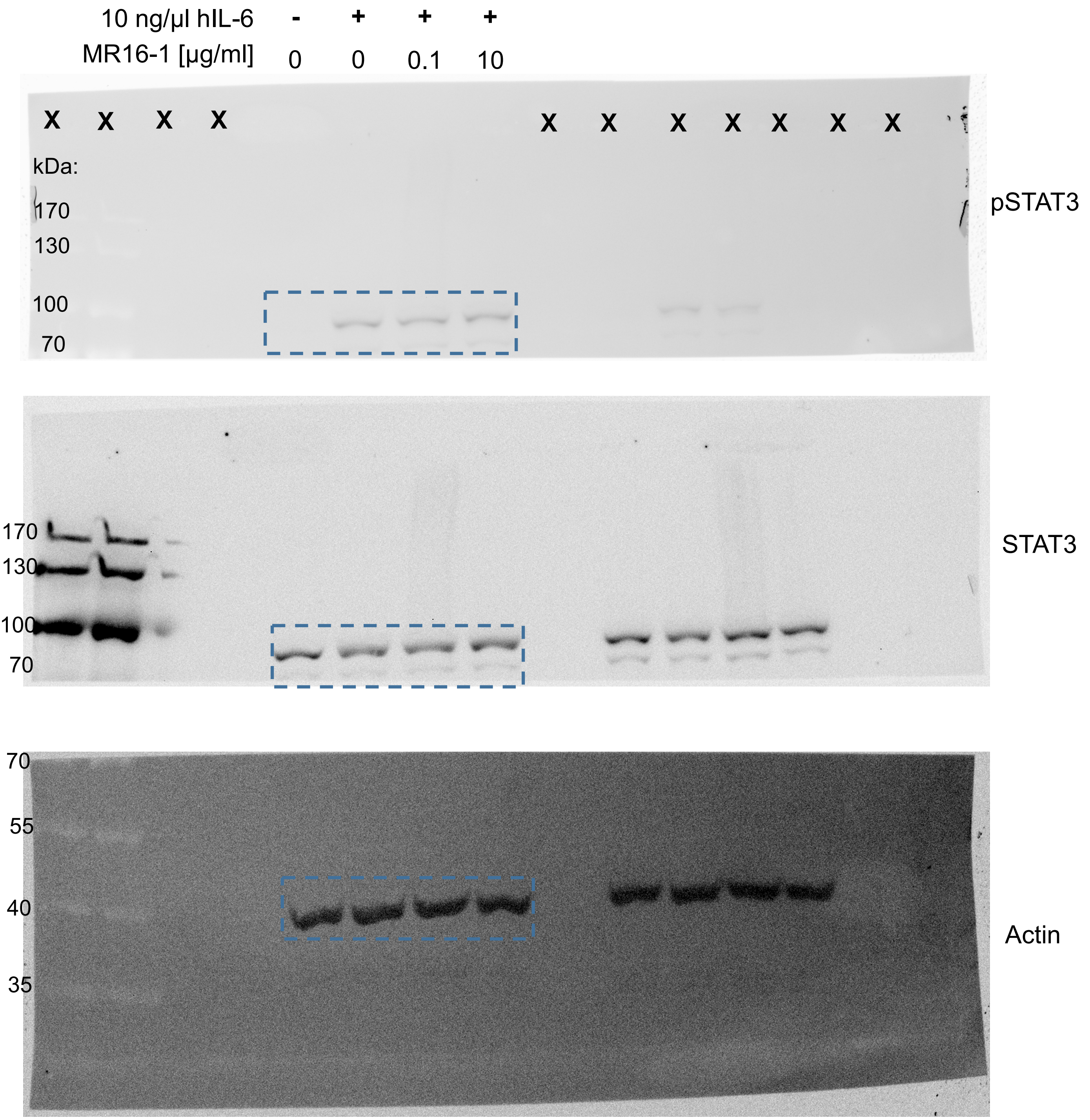

Detected with Alexa Fluor488- and Alexa Fluor647-conjugated antibodies using a ChemoStar ECL Imager (Intas)

Lanes not included in the figures are marked with an X

Figure 3D

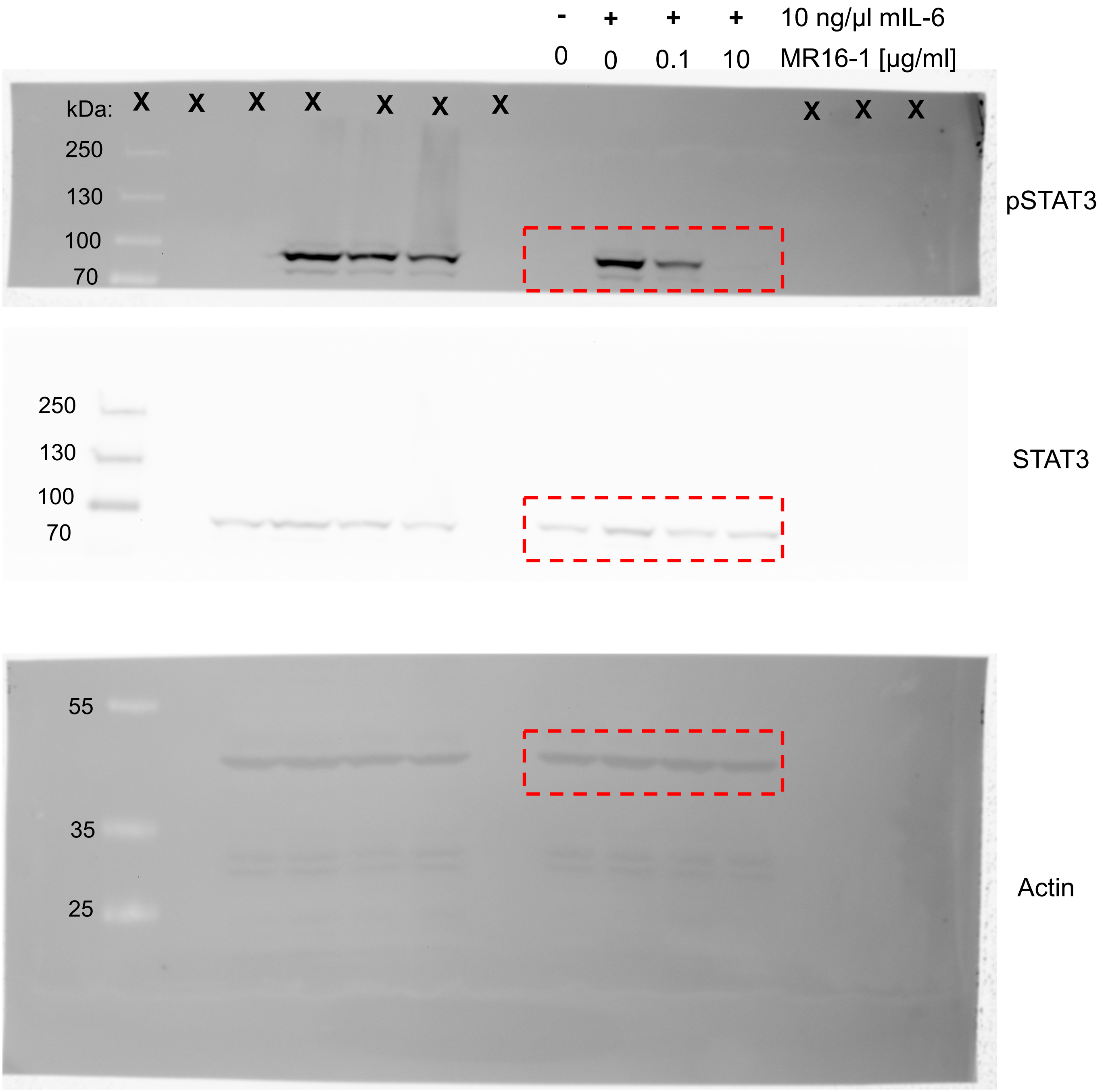

Detected with Alexa Fluor488- and Alexa Fluor647-conjugated antibodies using a ChemoStar ECL Imager (Intas)

Lanes not included in the figures are marked with an X

Figure 4A

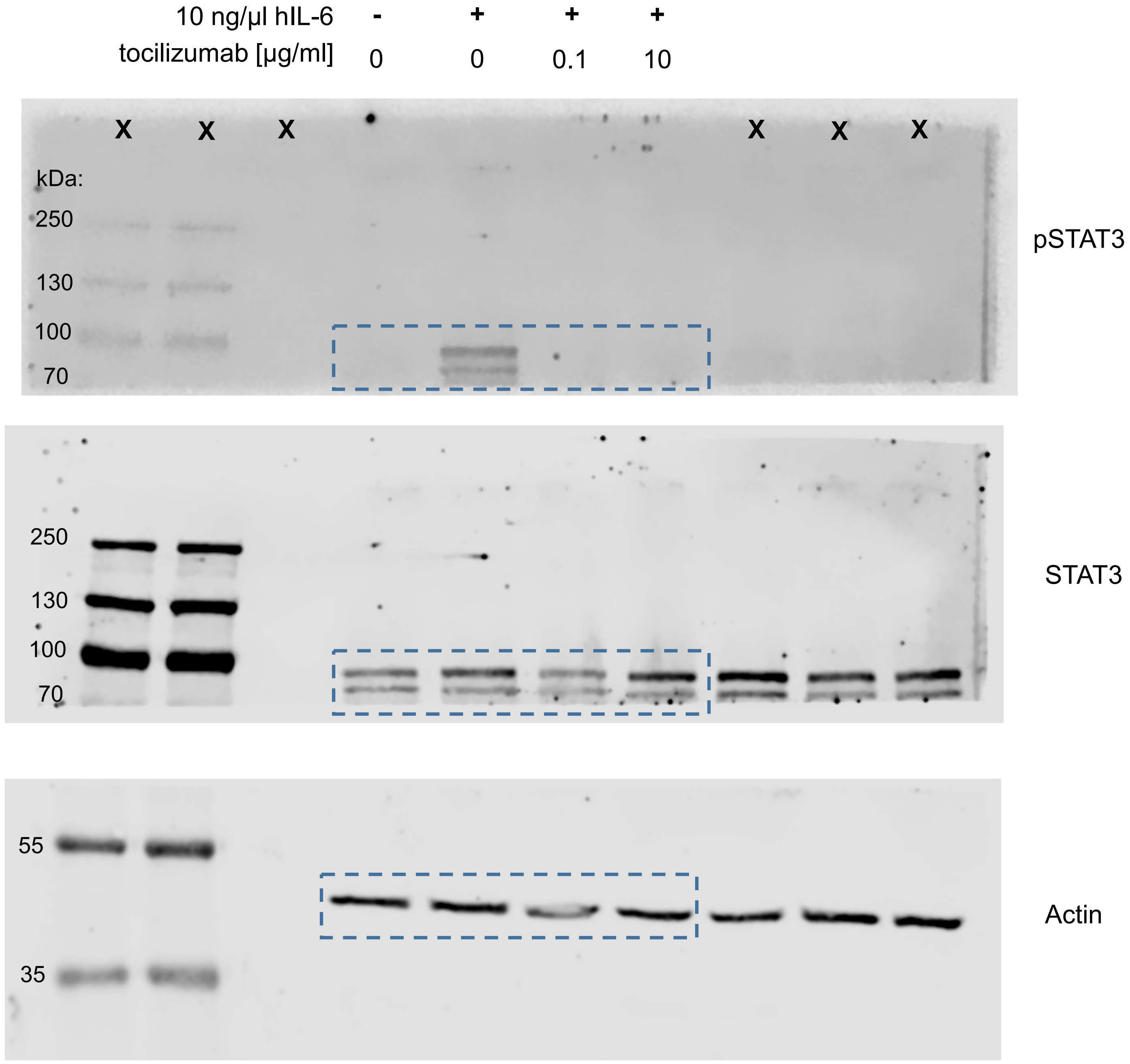

Detected with IRDye 680RD- and IRDye 800CW-conjugated antibodies using a Odyssey Fc Imager (LI-COR)

Lanes not included in the figures are marked with an X

Figure 4B

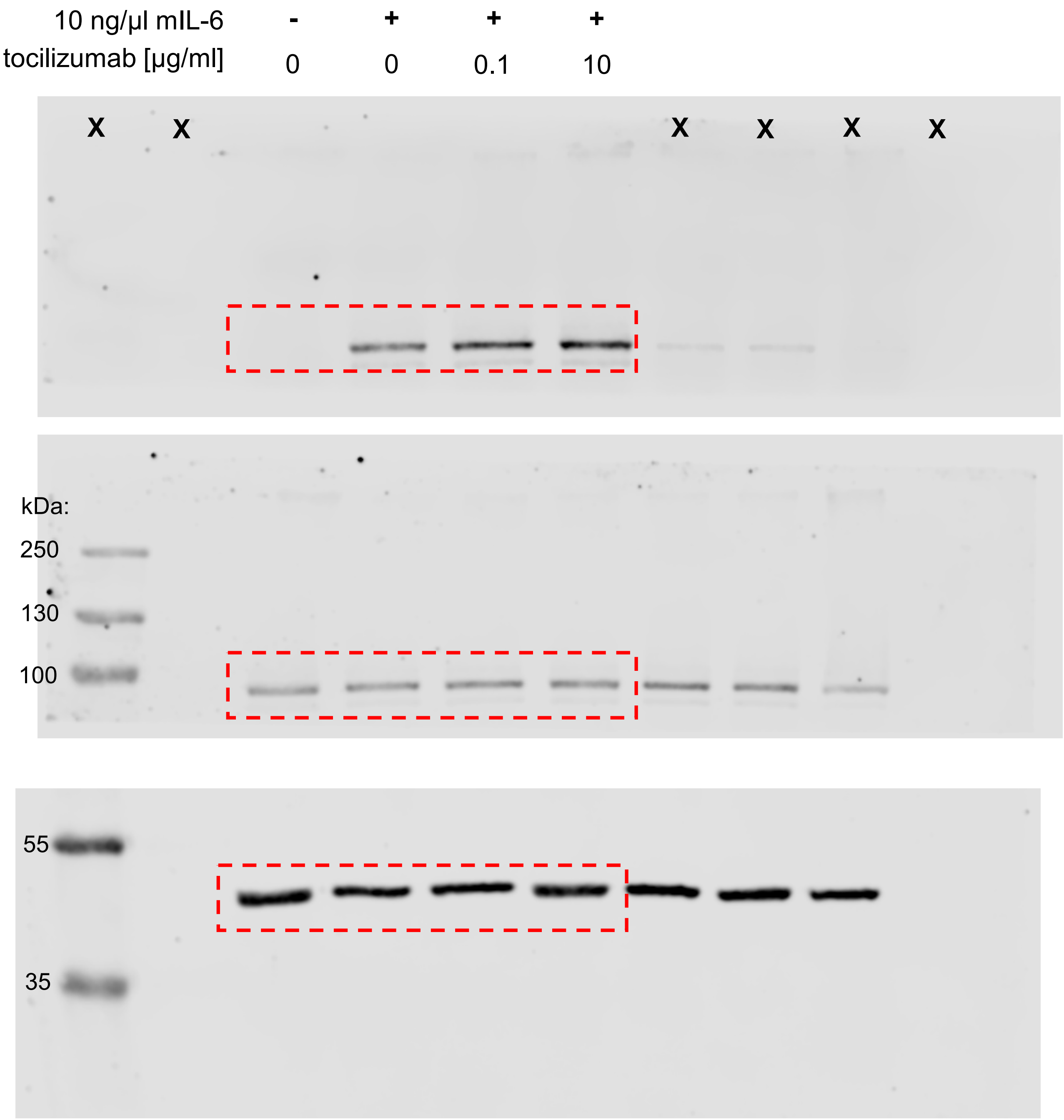

Detected with IRDye 680RD- and IRDye 800CW-conjugated antibodies using a Odyssey Fc Imager (LI-COR)

Lanes not included in the figures are marked with an X

Figure 4C

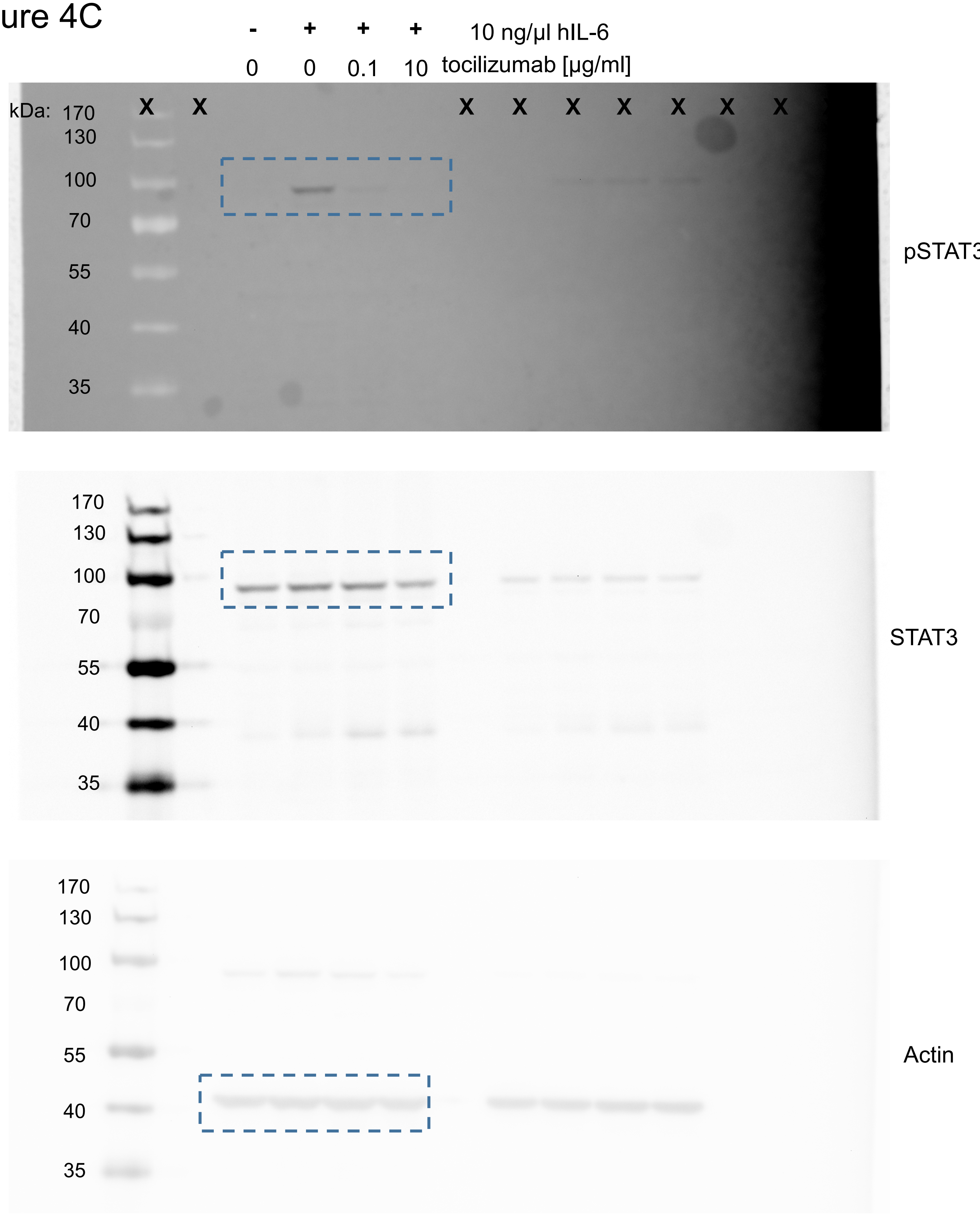

Detected with Alexa Fluor488- and Alexa Fluor647-conjugated antibodies using a ChemoStar ECL Imager (Intas)

Lanes not included in the figures are marked with an X

Figure 4D

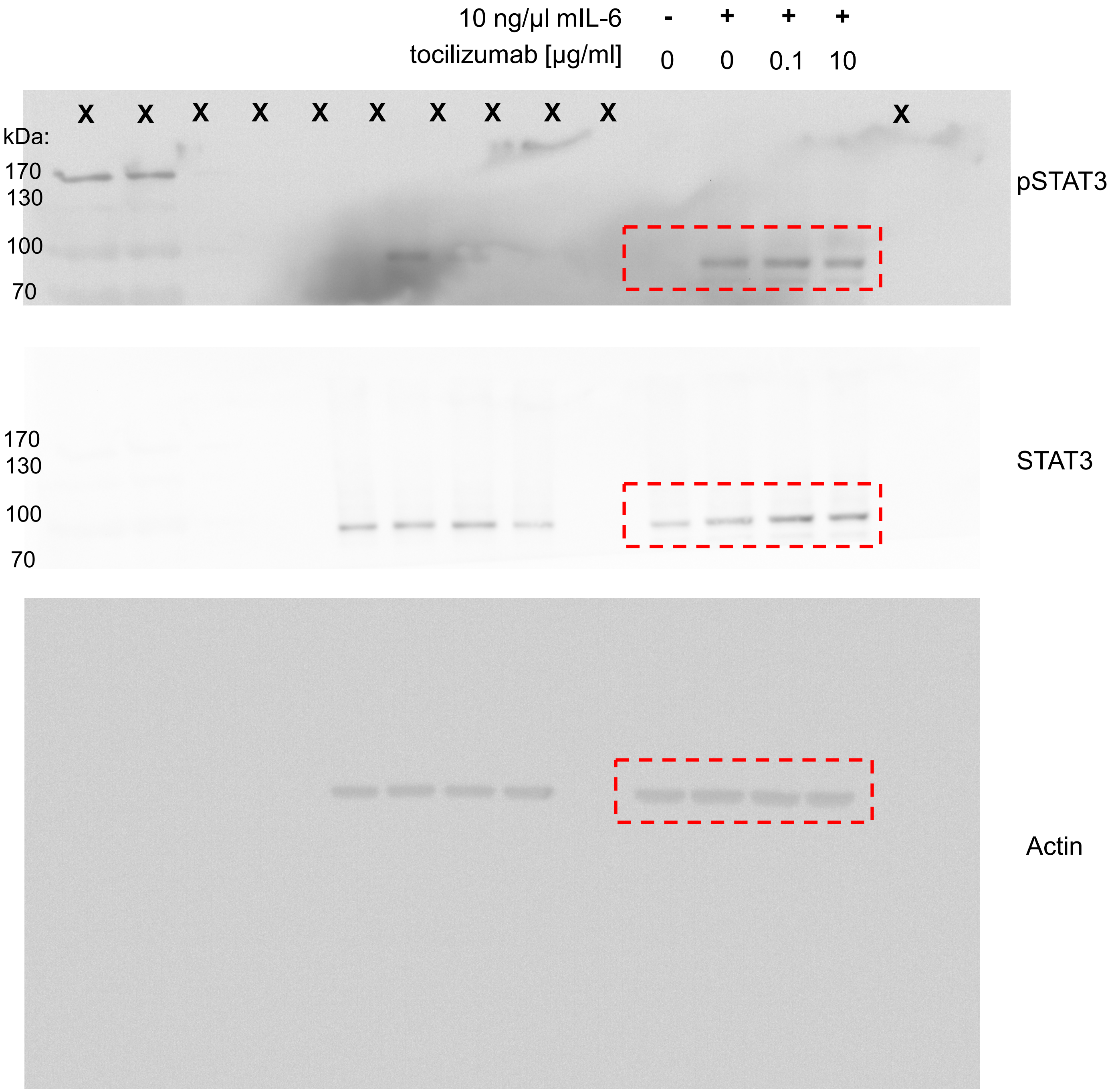

Detected with Alexa Fluor488- and Alexa Fluor647-conjugated antibodies using a ChemoStar ECL Imager (Intas)

Lanes not included in the figures are marked with an X
